# Functional Segregation of Epileptogenicity within the Human Amygdala

**DOI:** 10.1002/ana.78200

**Published:** 2026-04-01

**Authors:** Odile Feys, Julia Makhalova, Samuel Medina Villalon, Maria Fratello, Hugo Dary, Stanislas Lagarde, Francesca Pizzo, Agnès Trébuchon, Maxime Guye, Jean‐Philippe Ranjeva, Romain Carron, Didier Scavarda, Christian‐George Bénar, Bernard Giusiano, Fabrice Bartolomei

**Affiliations:** ^1^ APHM, Timone Hospital, Epileptology and Cerebral Rhythmology Marseille France; ^2^ Aix‐Marseille Université, INSERM, INS, Institut de Neurosciences des Systèmes Marseille France; ^3^ Aix Marseille Université, CNRS, CRMBM Marseille France; ^4^ APHM, Timone Hospital, CEMEREM Marseille France; ^5^ APHM, Timone Hospital, Functional and Stereotactic Neurosurgery Marseille France; ^6^ APHM Timone Enfant, Pediatric Neurosurgery and epilepsy surgery unit Marseille France

## Abstract

**Objective:**

In temporal lobe epilepsy (TLE), the amygdala in the epileptogenic network is underestimated compared to other regions such as the hippocampus. Recent advances in anatomical neuroimaging and stereoelectroencephalography (SEEG) signal analyses could help better understand the involvement of the different amygdala nuclei in the genesis of temporal lobe seizures.

**Methods:**

We retrospectively included 51 patients suffering from TLE who underwent SEEG over the past 5 years. The Virtual Epileptic Patient atlas with an integrated amygdala atlas was used to automatically localize SEEG contacts within the brain regions, including 9 amygdala nuclei. The Epileptogenicity Index (EI) and Connectivity Epileptogenicity Index (cEI) were computed on ictal SEEG recordings. We used a beta mixed model to evaluate the relative effects of amygdala nuclei, TLE subtypes, and lateralization of the epileptogenic zone on the epileptogenicity. We used the Wilcoxon rank sum test to study the associations between epileptogenicity level of distinct amygdala nuclei and ictal semiology (sensory, affective, cognitive, motor, and autonomic).

**Results:**

We observed higher epileptogenicity within the basolateral (BL) nucleus compared to other nuclei of the basolateral complex (lateral (LA), accessory basal (BM), and paralaminar (PL) nuclei) across all TLE subtypes. Regarding semiology, BL was more epileptogenic in patients with sensory phenomena and LA in patients with autonomic phenomena, while PL was less epileptogenic in patients with cognitive phenomena.

**Interpretation:**

Our findings disentangle the different epileptogenicity of amygdala nuclei in temporal lobe seizures. The observed epileptogenicity variance across amygdala nuclei can be explained by underlying neuronal and cytoarchitectural substrates. ANN NEUROL 20269999:n/a–n/a

Temporal lobe epilepsy (TLE) is the most frequent type of epilepsy in adults, and hippocampal sclerosis remains its main associated structural lesion in surgical cases.^1^ However, over the past 2 decades, advances in stereoelectroencephalography (SEEG) have profoundly reshaped our understanding of TLE pathophysiology. Rather than representing a uniform entity, TLE comprises several distinct anatomo‐functional subtypes that reflect the diversity of epileptogenic networks. These subtypes include mesial TLE (mTLE, mesiotemporal structures are epileptogenic, ie, the amygdala and/or the hippocampus, and/or the entorhinal cortex), lateral TLE (lTLE, neocortical structures are epileptogenic, mostly the superior temporal gyrus), mesio‐lateral TLE (mlTLE, both mesiotemporal and neocortical structures are epileptogenic), and temporal‐plus epilepsies (TLE+), the latter involving temporo‐insular, temporo‐frontal, or temporo‐occipital networks.^2^, ^3^, ^4^, ^5^, ^6^, ^7^, ^8^ This classification stems from the SEEG‐based quantifications of seizure onset and propagation, highlighting that seizures often emerge from distributed networks rather than isolated foci.^4^

In mTLE, while the hippocampus is a major epileptogenic structure, other structures such as the entorhinal cortex^9^, ^10^, ^11^ or the amygdala can also exhibit high epileptogenicity, initiating seizures consecutively or even simultaneously to the hippocampus,^3^, ^12^ especially in patients with normal magnetic resonance imaging (MRI).^4^

Amygdala enlargement (AE) is a well‐documented abnormality in patients with epilepsy. However, its role in the epileptogenic zone (EZ) is debatable, particularly in light of recent findings showing that AE is bilateral in one‐third of refractory focal epilepsy cases, and is associated, particularly for some specific nuclei, with the severity of psychiatric comorbidities.^13^ Noteworthy, TLE+ appears as the most frequent subtype of epileptogenic network in patients with AE.^14^ Furthermore, the amygdala has shown different effective connectivity patterns depending on whether it is a part of the EZ or not.^15^ The amygdala is composed of several distinct nuclei (Fig 1) that are anatomically connected to different brain structures and functionally implicated in various networks and often described as input vs. output nuclei according to a simplistic dichotomy.^16^, ^17^ Histopathological findings in surgical specimens demonstrated high variability of cytoarchitecture between nuclei.^18^ While their delineation was initially based on histology,^19^ recent advances in neuroimaging allowed for *in vivo* morphological characterization of the main amygdala nuclei.^13^

**FIGURE 1:**
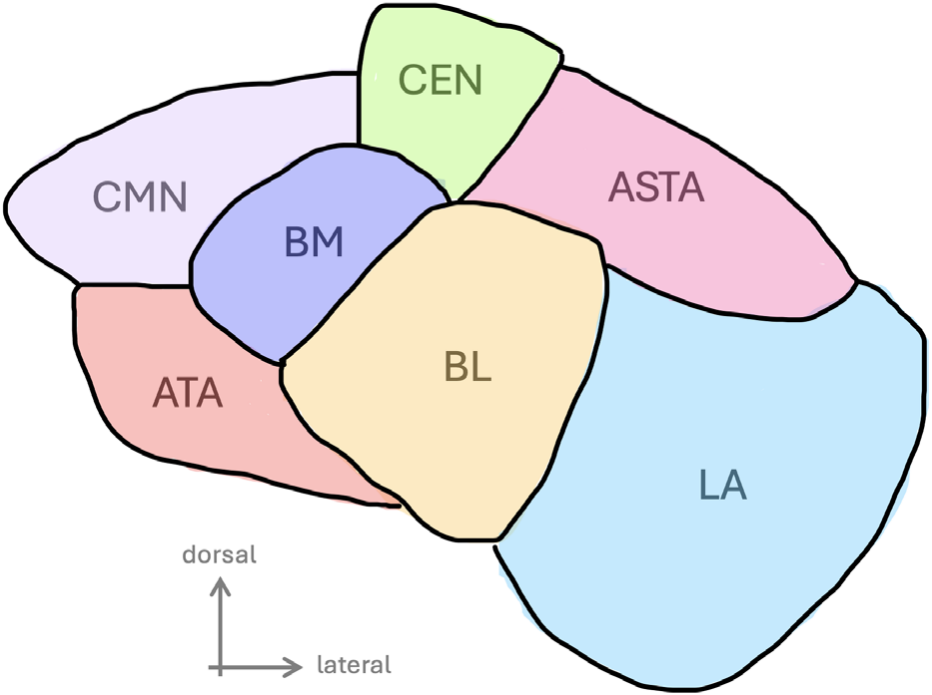
Schematic view of the left amygdala. This coronal slice through the left amygdala highlights 7 of the 9 nuclei explored in the study: the lateral nucleus (LA, light blue), the basolateral nucleus (BL, yellow), the accessory basal nucleus (BM, dark blue), the corticomedial nucleus (CMN, lila), the central nucleus (CEN, green), the periamygdaloid cortex (ATA, red), the amygdalo‐striatal transition area (ASTA, pink). The anterior amygdaloid area (AAA) and the paralaminar nucleus (PL) are not visible on the slice. (Schematic view based on ^13^ and ^25^.) [Color figure can be viewed at www.annalsofneurology.org]

Due to their structural and functional specificity, various amygdala nuclei may play different roles in the epileptogenic networks^3^ depending on the TLE subtype.^2^ Taking advantage of a fully automated, atlas‐based amygdala and nuclei segmentation pipeline^13^ now applicable on conventional 3 T MRI, it became possible to localize each SEEG electrode contact within the amygdala with respect to its distinct nuclei, and precisely estimate the epileptogenicity of each sampled nucleus.

This study aimed at (1) comparing the amygdala involvement in different types of TLE, (2) investigating the epileptogenicity profiles of different amygdala nuclei, (3) assessing whether epileptogenicity of distinct amygdala nuclei correlates with that of the other mesial temporal structures (the rhinal cortex and the hippocampus) in mTLE, and (4) studying the links between the epileptogenicity of specific amygdala nuclei and ictal semiology.

## Methods

### Inclusion Criteria and Clinical Data Collection

We retrospectively included all consecutive patients aged >12 years with refractory TLE who underwent an SEEG recording in the Department of Epileptology of la Timone Hospital, Marseille, between January 2020 and December 2024. SEEG was performed as a part of routine presurgical assessment following a noninvasive evaluation (medical history, neurological examination, neuropsychological testing, scalp video‐EEG, magnetoencephalography, 3 T MRI, ^18^[F]fluorodeoxyglucose (FDG)–positron emission tomography (PET) in all patients, 7 T MRI in some patients). Sixty patients matched the inclusion criteria. Five patients who underwent resection of mesial temporal structures prior to SEEG, and 4 others without amygdala sampling, were excluded. The remaining 51 patients constituted our final cohort. All patients gave their informed written consent to participate in the study, which was approved by Assistance Publique—Hôpitaux de Marseille (registration number of health data access portal, PADS 4SC2RS).

Seizure semiology was collected from patients' medical files based on patients' descriptions and physicians' reports. Semiological features were described based on the descriptors for focal seizures according to the International League Against Epilepsy (ILAE) glossary of seizure semiology adopted by the ILAE 2025 classification of epileptic seizures^20^, ^21^ as elementary motor, complex motor, sensory, cognitive (and language), autonomic, and affective phenomena. The full semiological repertoire of symptoms during the seizure was used for the analyses. For the purpose of statistical analysis in the present study, we merged elementary and complex motor phenomena into a single category of motor phenomena. By the same way, to enable uniformity of data for statistical analysis across the whole cohort, the described semiological features were used as categorical variables and reported as present or absent, regardless their time of occurrence during the seizure sequence, as previously reported for hyperkinetic seizures in.^22^

### SEEG Recordings

Implantations of intracerebral multiple contact electrodes (10–18 contacts with length 2 mm, diameter 0.8 mm, inter‐contact distance 1.5 mm; Alcis, France) were performed stereotactically using a robot‐assisted procedure. The implantation scheme in each patient was based on the anatomo‐electro‐clinical hypotheses of the EZ formulated from their noninvasive data and was therefore variable between patients. Post‐implantation computed tomography (CT) and MRI were performed to check the electrode positions and exclude intracranial bleeding. SEEG was recorded on a 256‐channel Natus system, sampled at 1024 Hz, and digitized at 16‐bit resolution without a digital filter. Two hardware filters were enabled in the acquisition system: a high‐pass filter (cutoff frequency equal to 1 Hz at −3 dB) and an antialiasing low‐pass filter (cutoff at 340 Hz).

### MRI Acquisition

MRI was performed in all patients on a 3 T Magnetom Verio or Magnetom Vida MR system (Siemens, Erlangen, Germany) using a 32‐channel phased‐array head coil. The protocol included a 3‐dimensional T1‐weighted magnetization‐prepared rapid gradient echo (3D‐MPRAGE) sequence (echo time [TE]/repstition time [TR]/inversion time [TI] = 3/2300/900 ms, 160 sections, 256 × 256 mm^2^ field of view [FOV], 256 × 256 matrix, spatial resolution = (1.0 × 1.0 × 1.0) mm^3^) for high‐resolution anatomical imaging.

### Amygdala Nuclei Segmentation and Localization of SEEG Contacts

First, volumetric segmentation and cortical surface reconstruction from the patient's 3D‐MPRAGE data were obtained using the recon‐all pipeline of the FreeSurfer (http://surfer.nmr.mgh.harvard.edu)^23^ software, and the virtual epileptic patient (VEP) atlas for automated cortical and subcortical brain parcellation (https://ins-amu.fr/vep-atlas)^24^ was computed.

Automated, atlas‐based volumetric segmentation of the amygdala and its nuclei was performed using patient's 3D‐MPRAGE images and a method previously described in^13^ that combines the segmentation of the whole amygdala based on the in‐house developed 7TAMI atlas^14^ with the segmentation of the 9 amygdala nuclei based on the atlas by Tyszka and Pauli^25^ on the 7TAMI template^26^: anterior amygdaloid area (AAA), amygdalo‐striatal transition area (ASTA), periamygdaloid cortex (ATA), basolateral nucleus (BL), accessory basal nucleus (BM), central nucleus (CEN), corticomedial nucleus (CMN), lateral nucleus (LA), paralaminar nucleus (PL). These parcellations were projected and merged into the VEP atlas in the patient‐specific MRI space.

**FIGURE 2:**
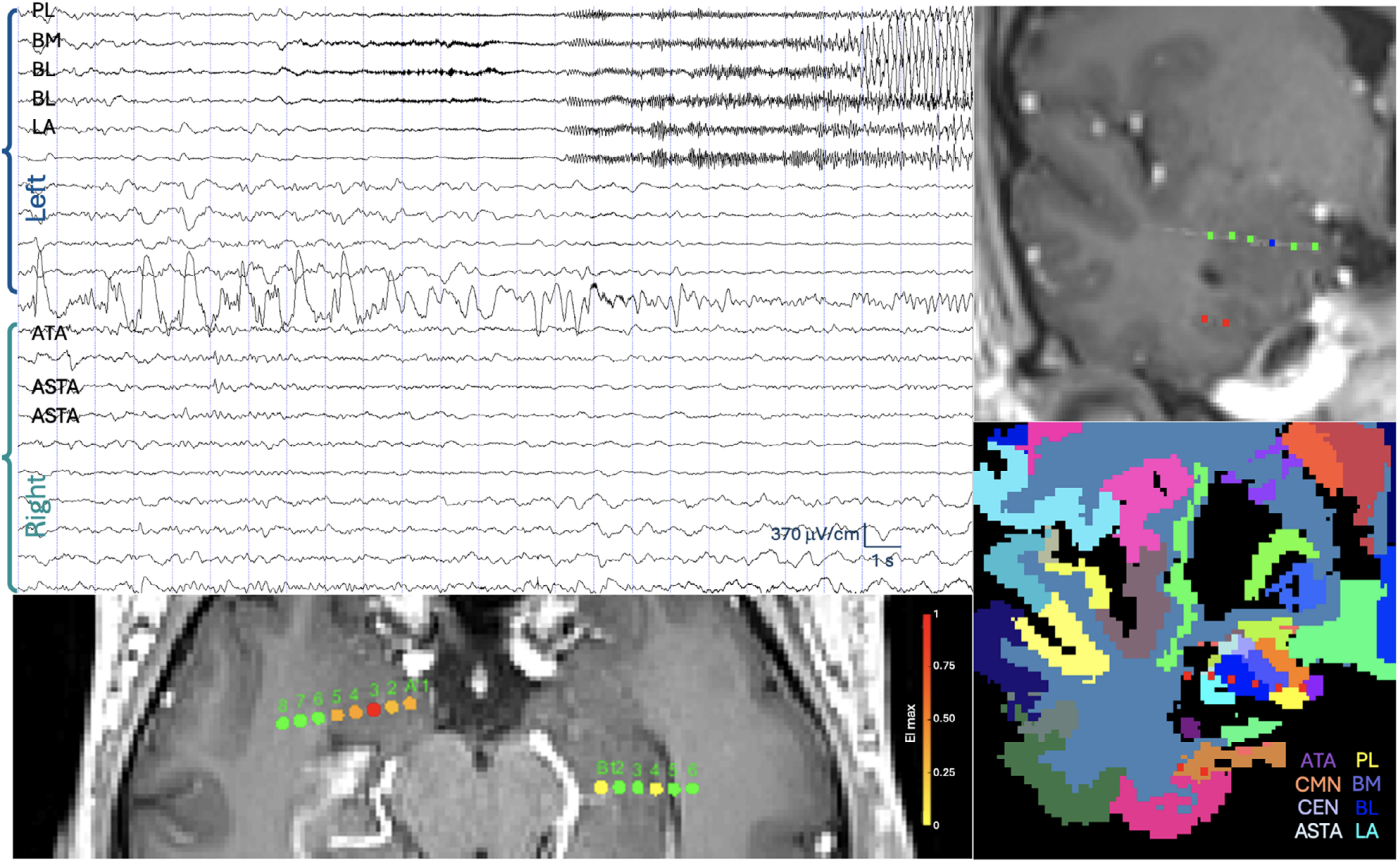
Example of the amygdala involvement in a left medial temporal seizure. A 36‐year‐old right‐handed patient with magnetic resonance imaging (MRI) ‐negative, drug‐resistant focal epilepsy underwent stereoelectroencephalography (SEEG) as part of routine presurgical work‐up, with the main hypothesis of the left temporal mesial‐basal epileptogenic zone (EZ). A left‐predominant bilateral implantation was performed (15 left and 2 right electrodes) to delineate the EZ, assess its relationships with functional areas, and evaluate the contribution of contralateral mesial temporal structures to the EZ network extent and functional prognosis. Right: automated visualization and labeling of electrodes in patient's anatomy using the Gardel software. Top right. Coronal view of the patient's 3D T1 MRI (displayed in neurological convention) showing localization of the A' electrode contacts within the patient's left amygdala (selected contact A'3 (left BL) is lighted in blue, other A' contacts in green). Bottom right. A' electrode contacts (red dotes) overlayed onto the amygdala nuclei parcellation (Tyszka and Pauli atlas merged with the virtual epileptic patient [VEP] atlas) projected in the patient's MRI space. Abbreviations: ASTA = amygdalo‐striatal transition area; ATA = periamygdaloid cortex; BL = basolateral nucleus; BM = accessory basal nucleus; CEN = central nucleus; CMN = corticomedial nucleus; LA = lateral nucleus; PL = paralaminar nucleus. Top left. The 25‐seconds ictal SEEG signal dataset focusing on the contacts within the left and the right amygdala highlighting 6 sampled amygdala nuclei (left PL, BM, BL, and LA; right ATA and ASTA). Bottom left. Axial view of the patient's 3D T1 brain MRI (displayed in neurological convention using 3D‐Viewer) highlighting the A' electrode targeting the left amygdala, and the B electrode targeting the right hippocampus, with graphical representation of normalized Epileptogenicity index (EI) ^4^ values (color scale from green (EI = 0) to red (EI = 1) for intracranial contacts with the lowest to the highest epileptogenicity). Following epileptogenicity values were obtained across the sampled amygdala nuclei in this patient: EI values: PL, 0.32; BM, 0.27; BL, 1; LA, 0.31; ATA, 0; ASTA, 0.002; Connectivity Epileptogenicity Index (cEI) values: PL,0.33; BM, 0.35; BL, 1; LA, 0.35; ATA, 0; ASTA, 0.002. [Color figure can be viewed at www.annalsofneurology.org]

Then, co‐registration of the MPRAGE images with the post‐implantation CT images was performed. The SEEG electrode contacts (represented by their geometric center) were automatically localized, labeled in patient‐specific MRI space and projected into the VEP atlas with integrated amygdala nuclei segmentation to assign to each SEEG electrode contact its associated brain region, including the 9 amygdala nuclei, using GARDEL software (Fig 2, https://meg.univ-amu.fr/doku.php?id=epitools:gardel).^27^ In the present study, we used each pair of contiguous SEEG contacts located in the gray matter as a region of interest (ROI). If 2 adjacent contacts were located in different regions (eg, different amygdala nuclei), the ROI topography was defined by the anatomical location of the geometric mean of the distance between the centers of these contacts.

### Epileptogenicity Measures and Definition of TLE Subtypes

SEEG‐signal analyses were performed using AnyWave software (https://gitlab-dynamap.timone.univ-amu.fr/anywave/documentation/-/wikis/home),^28^ in a bipolar montage including all contacts within the gray matter that was automatically generated using GARDEL. Two ictal epileptogenicity markers, the Epileptogenicity Index (EI, the ratio of high frequency activities relative to low frequency activities and the timing of involvement of each brain region)^4^ and the Connectivity Epileptogenicity Index (cEI, combining the original EI and a directed functional connectivity measure (“out‐degree”) in a single quantity and suitable for seizure‐onset patterns without low‐voltage fast activity),^29^ were computed simultaneously using a dedicated Matlab plug‐in (https://meg.univ-amu.fr/doku.php?id=plugins:ei).

The regions involved in the EZ network were identified based on the validated EI/cEI thresholds^30^ (EI ≥ 0.40 and/or cEI ≥0.65). These epileptogenicity measures were represented within the patient's anatomy using 3DViewer (https://meg.univ-amu.fr/doku.php?id=epitools:3dviewer) (see Fig 2 for an example with different amygdala nuclei).^27^ All analyses, either at the level of the whole amygdala or at the level of single nuclei, or other explored regions, were performed using the maximal normalized EI and the maximal cEI values for each bipolar channel unless explicitly specified otherwise. If a region (or an amygdala nucleus) was sampled by 2 or more bipolar contacts, the maximal EI/cEI values obtained for this region (nucleus) were used.

TLE subtypes were defined based on the topography of EZ, with mTLE only involving epileptogenic mesial temporal regions, lTLE only lateral temporal regions, mlTLE both mesial and lateral temporal regions and TLE+ temporal and extratemporal regions.

### Description of Seizure Onset Patterns

We classified the seizure onset patterns (SOP) in the amygdala using visual and time‐frequency analysis according to the methodology described elsewhere.^31^, ^32^ These patterns include: (A) low‐voltage fast activity (LVFA); (B) preictal spiking followed by LVFA; (C) burst of polyspikes followed by LVFA; (D) slow wave or baseline shift followed by LVFA; (E) rhythmic slow spikes; (F) theta/alpha sharp activity; (G) beta sharp activity; (H) delta‐brush.^31^

### Statistical Analyses

All patients were included in the statistical analyses, with amygdala epileptogenicity treated as a continuous variable (EI and cEI values) unless stated otherwise below. We first assessed the prevalence of sampling of each of the 9 main amygdala nuclei in the present cohort. Due to the small number of samples for some nuclei, the epileptogenicity of individual nuclei could not be directly statistically compared between the nuclei. Therefore, we regrouped the nuclei into 3 subgroups based on their anatomy, function and cytoarchitecture to perform statistical analyses, that is, (1) BL, BM, PL, and LA; (2) AAA, ATA, and ASTA; and (3) CMN and CEN. We then compared the epileptogenicity of individual nuclei of the basolateral complex (BL, BM, PL, and LA), which were the most sampled in our cohort. The distribution of the dependent variables EI and cEI following a Beta law with parameters alpha <1 and beta <1, we applied a Beta mixed model regression (R package glmmTMB^33^) on data from these subgroups and on data from the basolateral complex to compare their levels of epileptogenicity and reveal potential epileptogenic heterogeneity within the amygdala. We included in our models patients as a random effect and as fixed effects the type of TLE (mTLE, lTLE, mlTLE vs. TLE+) and hemispheric lateralization with regard to the EZ (ipsilateral vs. contralateral, left and right hemispheres were considered as ipsilateral in patients with bilateral TLE) to examine whether the potential heterogeneity in amygdala epileptogenicity reflects these features. Then, we investigated possible associations between the epileptogenicity level of distinct amygdala nuclei (EI or cEI values, quantitative variable) and ictal semiology (sensory, affective, cognitive, motor, autonomic – present or absent, categorical variable) using Wilcoxon rank sum test. Lastly, we focused on mTLE and investigated correlations between the epileptogenicity levels of different mesial temporal structures including distinct amygdala nuclei, the rhinal cortex, and the hippocampus using Spearman test. A *p*‐value <0.05 was considered as significant. We also performed linear mixed models on EI values of each nucleus of the basolateral complex ipsilateral to the EZ, with fixed effects from epilepsy duration^34^ and etiology.

## Results

### Patients' Characteristics and SEEG Amygdala Sampling

Demographic and clinical data of the 51 included patients (27 females/24 males) are detailed in Table 1. In brief, our population involved 22 patients with mTLE, 16 patients with TLE+, 7 patients with lTLE, and 6 patients with mlTLE.

**TABLE 1:** Demographic and Clinical Data

Variable	Result
Age at SEEG (mean ± standard deviation)	32 ± 11 years
Age at epilepsy onset (mean ± standard deviation)	18 ± 10 years
Epilepsy duration (mean ± standard deviation)	14 ± 10 years
Etiology	Unknown 21/51 Structural 30/51 Focal cortical dysplasia 6/30 Post‐traumatic sequelae 6/30 Hippocampal sclerosis 5/30 Heterotopia 5/30 Dysembryoplastic neuroepithelial tumour 4/30 Cavernoma 2/30 Vascular sequelae 1/30 Polymicrogyria with heterotopia 1/30
TLE subtypes (number of subjects)	mTLE 22/51 TLE+ 16/51 lTLE 7/51 mlTLE 6/51
Amygdala involved in EZ	36/51
EZ lateralization (epileptogenic amygdala lateralization)	Left 24/51 (14/36) Right 20/51 (15/36) Bilateral 7/51 (5/36)
Surgery	Open surgery 24/51 Laser interstitial thermal therapy 2/51
Postoperative outcome at 1‐year, Engel’	Engel 1–17/26 Engel 2–6/26 Engel 3–2/26 Engel 4–1/26
Ictal semiology *Ratio of patients with EI = 1 in the amygdala among those with described semiology (%)*	Sensory 32/51 *12/32 (38%)* Affective 23/51 *8/23 (35%)* Cognitive 37/51 *7/37 (19%)* Motor 33/51 *12/33 (36%)* Autonomic 27/51 *9/27 (33%)*

Abbreviations: EI = Epileptogenicity Index; EZ = epileptogenic zone; lTLE = lateral temporal lobe epilepsy; mlTLE = mesial‐lateral temporal lobe epilepsy; mTLE = mesial temporal lobe epilepsy; SEEG = stereoelectroencephalography; TLE = temporal lobe epilepsy; TLE+ = temporal‐plus epilepsy.

SEEG contacts were located within the 9 amygdala nuclei in various proportions, as reported in Table 2, the basolateral complex (ie, LA, BL, BM, and PL) being the most sampled compared to other nuclei, and ASTA, AAA, and CEN being the least sampled.

**TABLE 2:** Number of Patients with Sampling of Each Amygdala Nuclei

Amygdala Nuclei	Left	Right	Ratio of Epileptogenic Contacts / Contacts Ipsilateral to the EZ
Lateral nucleus (LA)	29	14	23/61 (38%)
Basolateral nucleus (BL)	32	16	28/54 (52%)
Accessory basal nucleus (BM)	14	4	7/16 (44%)
Paralaminar nucleus (PL)	17	7	14/25 (56%)
Periamygdaloid cortex (ATA)	5	3	3/7 (43%)
Anterior amygdaloid area (AAA)	2	0	1/2 (50%)
Amygdalo‐striatal transition (ASTA)	2	1	0/3 (0%)
Corticomedial nucleus (CMN)	6	1	2/6 (33%)
Central nucleus (CEN)	2	0	1/3 (33%)

Abbreviations: EZ, epileptogenic zone.

### Global Epileptogenicity across Brain Regions and Amygdala SOP

**FIGURE 3:**
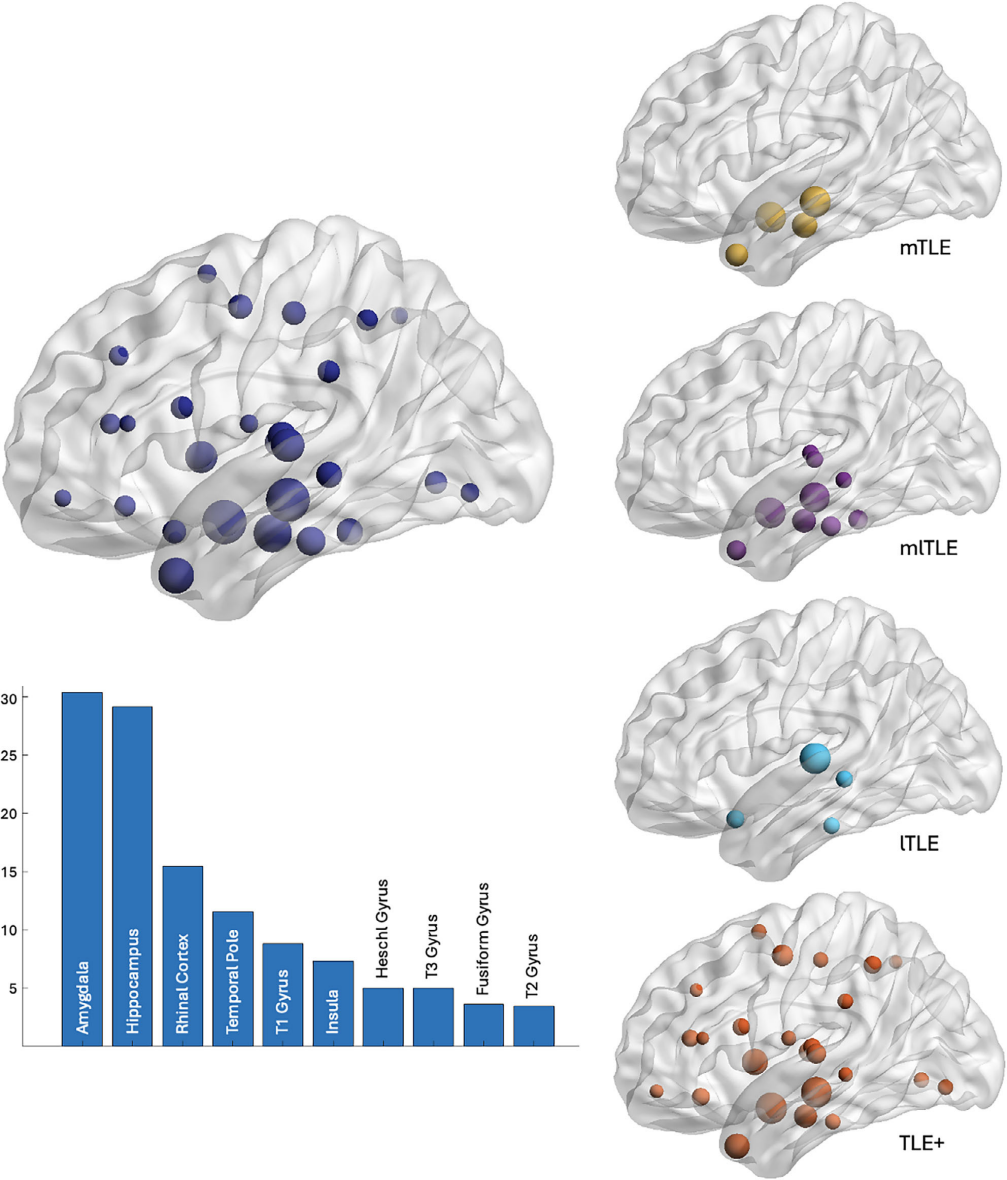
Cumulative epileptogenicity values across epileptogenic regions in temporal lobe epilepsy. Left. Summed normalized Epileptogenicity Index (EI) values above the 0.4 cutoff across brain regions in 51 patients with temporal lobe epilepsy (TLE, dark blue), represented on a template brain map (top), and the 10 epileptogenic regions disclosing the highest summed EI values on the bar plot (bottom). Right. Summed EI values above the 0.4 cutoff across brain regions in patients with mesial TLE (mTLE, yellow), mesial‐lateral TLE (mlTLE, purple), lateral TLE (ltle, light blue), and temporal‐plus (TLE+, orange) subtypes. Brain maps are built with BrainNet Viewer using 90 AAL nodes.^71^ [Color figure can be viewed at www.annalsofneurology.org]

**FIGURE 4:**
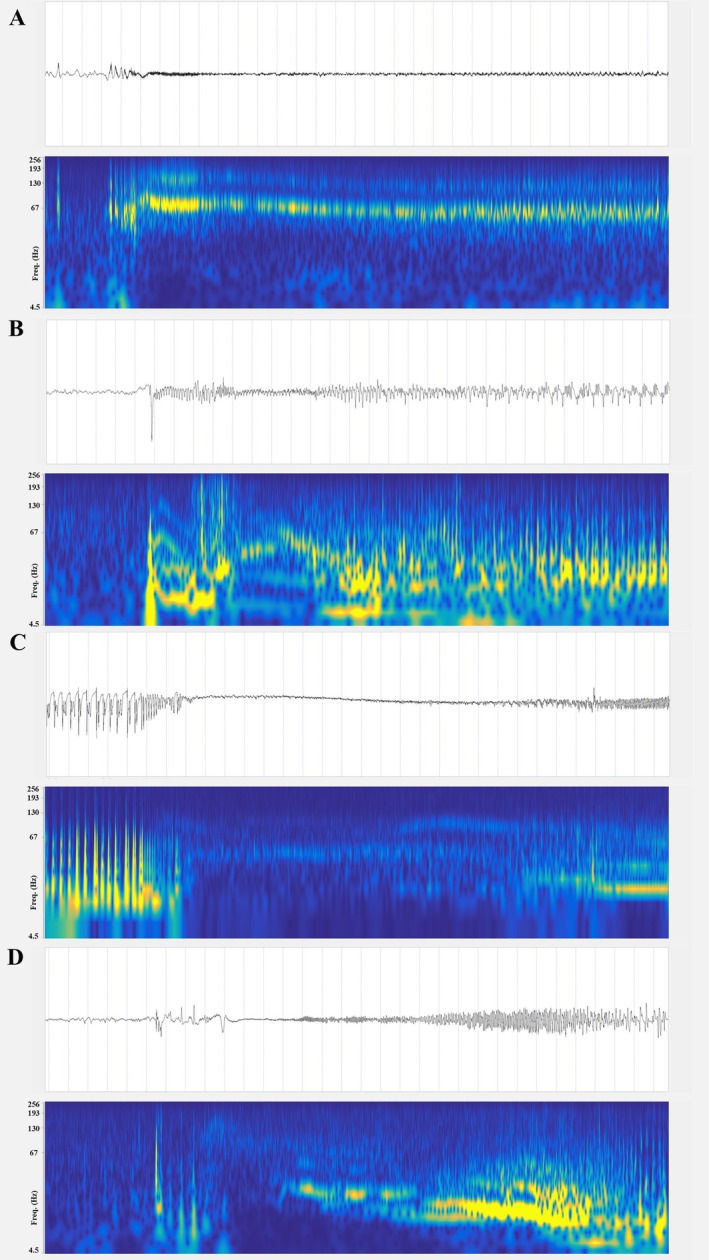
Seizure onset patterns of the amygdala. (A) Low‐voltage fast activity (LVFA). (B) Rhythmic theta pattern. (C) Preictal spiking followed by LVFA. (D) Bursts of polyspikes followed by LVFA. Top of each panel shows the temporal course and bottom the time frequency analysis. Dotted lines at the top of each panel give the temporal scale (1 second between 2 lines). [Color figure can be viewed at www.annalsofneurology.org]

In this TLE cohort, the amygdala and the hippocampus were the most epileptogenic structures compared with other brain regions (Fig 3). In mTLE, mlTLE, and TLE+, amygdala and hippocampus remained the most epileptogenic brain areas, while the superior temporal gyrus was the most epileptogenic brain area in lTLE. Unsurprisingly, the rhinal cortex and the temporal pole disclosed high epileptogenicity in our cohort, and the insula was the most epileptogenic extratemporal brain region. The amygdala disclosed the maximal epileptogenicity values based on the EI (EI = 1) in 17/51 patients (33%, Table 1). Four additional patients showed maximal epileptogenicity in amygdala as estimated by the cEI (cEI = 1). Among these 21 patients with maximal epileptogenicity in the amygdala, the SOP was represented by LVFA in 11 patients, rhythmic theta activity in 5 patients, preictal spiking followed by LVFA in 3 patients, and bursts of polyspikes followed by LVFA in 2 patients (Fig 4).

### EI Analysis across Nuclei

**FIGURE 5:**
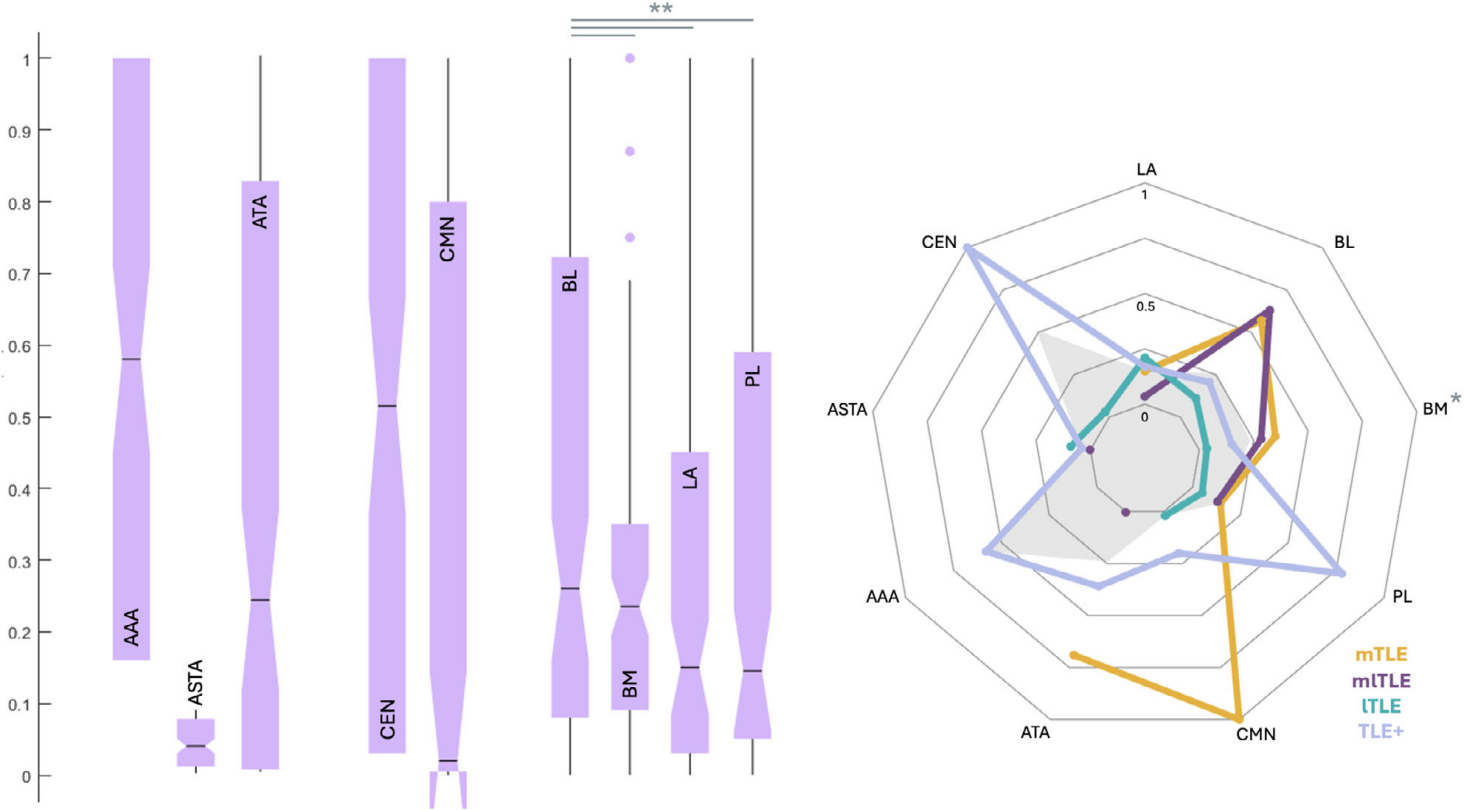
Distribution of Epileptogenicity Index (EI) values according to the amygdala nuclei and the subtypes of temporal lobe epilepsy. Left. Box plots showing distributions of normalized EI values across the sampled amygdala nuclei (lila). Black horizontal line: median value, color plot: interquartile range, black vertical line: distribution, color dots: outliers. Right. Radar plots of median EI values per each amygdala nucleus depending on the temporal lobe epilepsy (TLE) subtype (medial TLE, yellow; medial‐lateral TLE, purple; lateral TLE, turquoise; TLE+, light blue) and across all patients (grey shape). ** for significant differences (*p* < 0.05) and * for trend (*p* < 0.1). [Color figure can be viewed at www.annalsofneurology.org]

The distribution of EI values across sampled amygdala nuclei is shown in Fig 5. There was a considerable interindividual variability in EI values, resulting in very broad distributions for most nuclei. However, all nuclei except the ASTA could be involved in the EZ network (EI values ≥ 0.4) at the individual level in some patients. In the whole TLE cohort, the highest median EI values were observed in the AAA, CEN and BL. Various patterns of epileptogenicity were shown depending on the TLE subtypes, with higher median EI values in BL and CMN in the mTLE, whereas these values were maximal in CEN and PL in TLE+ (Fig 5).

The comparison between the 3 subgroups of nuclei (1: BL, BM, PL, LA; 2: AAA, ATA, ASTA; 3: CMN, CEN) showed no significant differences in epileptogenicity levels, while the analysis within the basolateral complex demonstrated significantly higher EI values in BL compared to LA, BM, and PL (*p* = 0.047). Additionally, there was a trend for the higher EI values in BM and in mTLE compared to other nuclei and TLE subtypes (*p* = 0.076), see Supplementary Figure S1.

Focusing on patients suffering from mTLE, we investigated correlations of epileptogenicity levels between different mesial temporal structures. We observed a strong correlation between the rhinal cortex and the hippocampus (ρ = 0.49, *p* < 0,001) and a significant correlation between LA and BL (ρ = 0.35, *p* < 0,05).

### Connectivity Epileptogenicity Index Analysis across Nuclei

**FIGURE 6:**
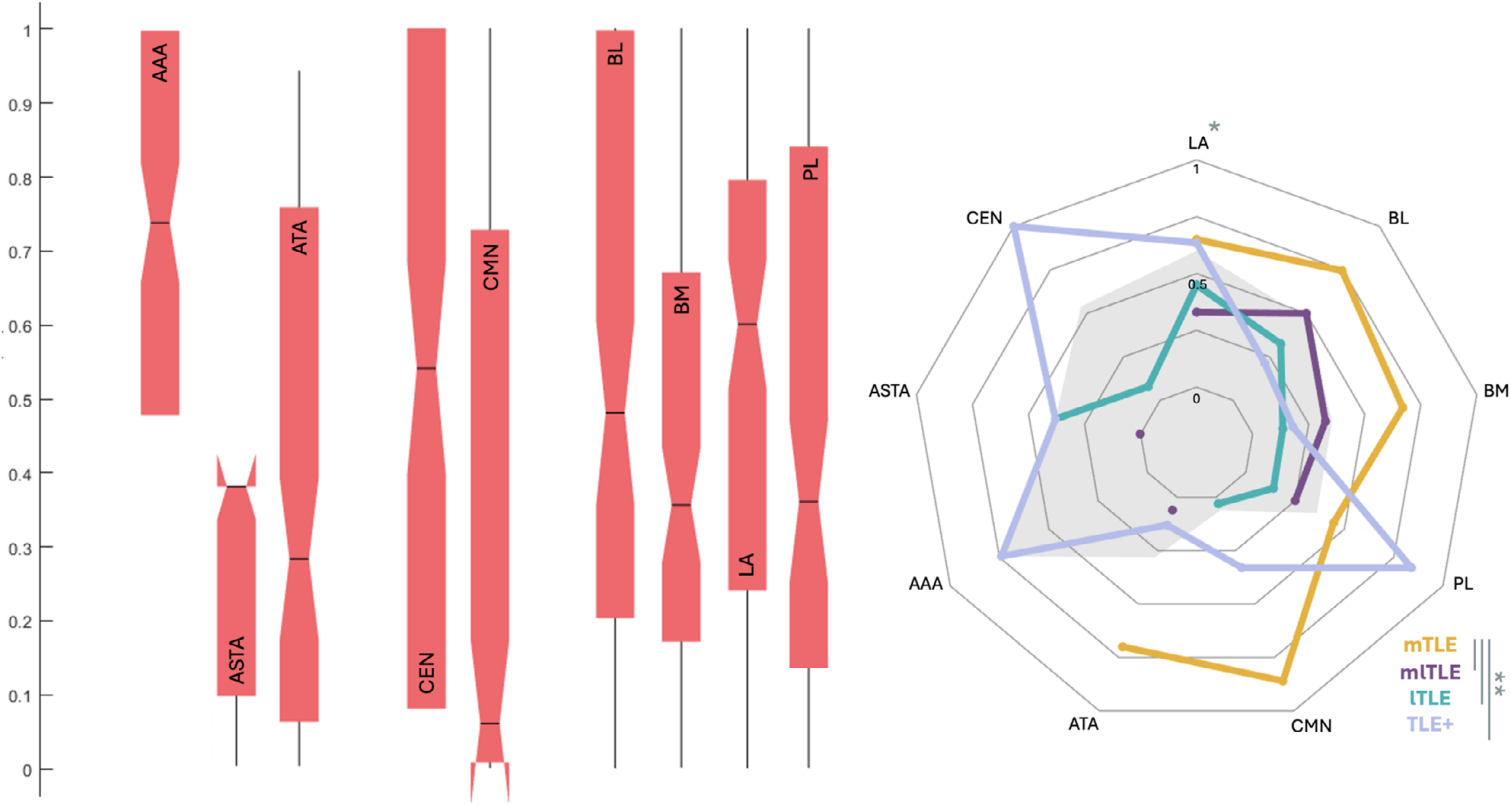
Distribution of Connectivity Epileptogenicity Index (cEI) values according to the amygdala nuclei and the subtypes of temporal lobe epilepsy. Left. Box plots showing distributions of cEI values across the sampled amygdala nuclei (red). Right. Radar plots of median cEI values per each amygdala nucleus depending on the temporal lobe epilepsy (TLE) subtypes (medial TLE, yellow; medial‐lateral TLE, purple; lateral TLE, turquoise; TLE+, light blue) and across all patients (grey shape). ** for significant differences (*p* < 0.05) and * for trend (*p* < 0.1). [Color figure can be viewed at www.annalsofneurology.org]

Similarly to the EI, there was a great interindividual variability with large distributions of cEI values across the sampled nuclei, all but ASTA could belong to the EZ network (cEI≥0.65) at the individual level. Various epileptogenicity patterns depending on the TLE subtypes were observed (Fig 6). The beta mixed models by subgroups and in the basolateral complex showed no significant epileptogenicity differences between different amygdala nuclei. They showed higher cEI values in mTLE compared to lTLE, mlTLE, and TLE+ (*p* = 0.032) and higher values in the hemisphere ipsilateral to the EZ (*p* < 0.001). Additionally, there was a trend for higher cEI values in LA and in mTLE (*p* = 0.07), see Supplementary Figure S1.

Focusing on patients suffering from mTLE, we investigated the correlations between the epileptogenicity levels across the amygdala nuclei and other mesial temporal structures. In the same way as for the EI, we observed a strong correlation between the cEI values of the rhinal cortex and the hippocampus (ρ = 0.43, *p* < 0.01), and a significant correlation between LA and BL (ρ = 0.58, *p* < 0.01). Additionally, we found a correlation between PL nucleus and hippocampal cortex (ρ = 0.48, *p* < 0.05).

### Associations with Ictal Semiology

The distribution of EI across patients depending on their ictal semiology is reported in Supplementary Figure S2–S6. EI values were higher in BL (*p* = 0.013) in patients with ictal sensory phenomena compared to those without. In patients with ictal cognitive phenomena, EI values were lower in PL (*p* = 0.028). Overall, cognitive phenomena were the least related to a high amygdala epileptogenicity (19%) compared to other ictal semiology (33–38%) (Table 1).

The distribution of cEI across patients depending on ictal semiology is reported in Supplementary Figure S2–S6. CEI values were lower in PL (*p* = 0.025) and LA (*p* = 0.033) in patients with ictal cognitive phenomena. In patients with ictal autonomic phenomena, cEI values were higher in LA (*p* = 0.006).

### Effect from Clinical Variables

The linear mixed models built to explain epileptogenicity of the nuclei within the basolateral complex demonstrated:

- A significant effect of the etiology on LA epileptogenicity (*p* = 0.017; post‐hoc analysis of variance (ANOVA) with *p* = 0.009) with a main effect from focal cortical dysplasia,
- A significant effect of the etiology and epilepsy duration on BM epileptogenicity (*p* = 0.0015; post‐hoc ANOVA with *p* = 0.0013 and *p* = 0.039) with a main effect from focal cortical dysplasia and hippocampal sclerosis,
- No significant effect was found for the BL epileptogenicity (*p* = 0.15), and
- No significant effect was found for the PL epileptogenicity (*p* = 0.22).

## Discussion

This study aimed to precisely characterize the epileptogenicity profiles of the amygdala and its 9 main nuclei in refractory temporal lobe epilepsy in relation to the TLE subtypes and investigate their possible links with ictal semiology and the epileptogenicity of other mesial temporal structures.

The amygdala was one of the most epileptogenic brain areas in our cohort of 51 patients with TLE. Most nuclei showed a large variance of the EI and cEI values across subjects and between the TLE subtypes. Still, our main results were related to the epileptogenicity of the basolateral complex. BL was more epileptogenic than other nuclei in the whole TLE cohort, while BM and LA tended to be more epileptogenic in mTLE. Amygdala nuclei epileptogenicity was also related to the sensory, autonomic and cognitive components of the ictal semiology. In mTLE, epileptogenicity levels of PL nucleus and hippocampus were correlated.

### Amygdala Nuclei Sampling

The nuclei of the basolateral complex (LA, BL, BM, PL) were the best sampled across the whole TLE cohort. This is not surprising because of the larger volume of this complex compared to other parts of the amygdala^35^, ^36^ and its location along with the common orthogonal trajectory of the amygdalar SEEG electrode.^37^ SEEG investigation of other nuclei, in particular of CEN and CMN would require an oblique or a more dorsal additional trajectory,^17^ with subsequently a much smaller number of cases with available sampling in the present cohort. This justifies focusing on the nuclei of the basolateral complex for group comparisons.

### Epileptogenicity of Amygdala Nuclei

All 9 investigated nuclei showed highly variable epileptogenicity values across patients and TLE subtypes (Figs 5 and 6), with failure to demonstrate any significant difference in epileptogenicity levels between the 3 assessed subgroups of nuclei. This negative result could be biased by insufficient sampling of nuclei outside the basolateral complex.

Within the basolateral complex, the BL was found to be the most epileptogenic nucleus. The synaptic organization of its pyramidal neurons is similar to that of the neocortex.^38^ This higher BL epileptogenicity than other nuclei could be due to its ability to generate intrinsic gamma bursts.^39^ In BL, the pyramidal cells are subdivided into late‐firing and burst‐firing neurons (32), and the fast‐spiking interneurons are often spontaneously active at rest due to a more depolarized membrane potential.^40^ Gamma oscillations in BL lead to stronger activation than those in LA, and to higher modulation of the excitation in the BL nucleus.^41^ Moreover, the experimental ablation of glutamatergic neurons in the BL nucleus was shown to regulate epileptic seizures in TLE.^42^

We observed that epileptogenic structural lesions (focal cortical dysplasia [FCD] and hippocampal sclerosis) influenced LA and BM epileptogenicity, and that longer epilepsy duration increased BM epileptogenicity. Noteworthy, these lesions were located outside the amygdala, and the observed effects do not demonstrate the link between the amygdala epileptogenicity and morphology. Although rare cases of histologically confirmed FCD within the amygdala have been previously reported, AE represents a common finding in MRI‐negative drug‐resistant epilepsy.^13^, ^43^ Our previous study in a small cohort of TLE patients with AE showed no correlation between the amygdala volume and cEI values.^14^ The literature data are heterogeneous regarding the links between the amygdala nuclei volume and etiology or MRI‐lesional status. While Ballerini et al^43^ reported ipsilateral atrophy of the basolateral complex in TLE with hippocampal sclerosis, and medial nucleus hypertrophy in MRI‐negative cases, a recent study by our group found no significant associations between the amygdala nuclei volume and MRI‐visible lesions or etiology.^13^ Overall, these data suggest that the epileptogenicity profiles rather depend on the involved epileptogenic networks. The observed effect from the highly epileptogenic lesion or epilepsy duration on the epileptogenicity levels of the LA and BM nuclei, characterized by moderate epileptogenicity, suggest a possibility of kindling‐like effect on these nuclei from the epileptogenic temporal lobe structures outside the amygdala.

Compared to the EI, the cEI, which estimates epileptogenicity by integrating the EI together with a functional connectivity measure, showed globally similar results, and additionally demonstrated higher amygdala epileptogenicity in mTLE than in other TLE subtypes, which could be due to the increased coupling of the amygdala with other mesial temporal structures at seizure onset.^10^, ^44^

### SOP in the Amygdala

Seizure onset patterns similar to those previously described in cortical brain structures (24) were observed in the epileptogenic amygdala in the present study, ie, LVFA (see Figure 2), rhythmic theta activity, preictal spiking followed by LVFA, and bursts of polyspikes followed by LVFA. These results confirm that cortical and subcortical epileptogenic structures exhibit common electrophysiological signatures, particularly characterized by a limited and reproducible repertoire of SOP, as previously described for the thalamus.^45^

### Epileptogenicity of Amygdala Nuclei Depending on TLE Subtypes

The epileptogenicity profiles of investigated nuclei (Figs 5 and 6) were concordant with the respective TLE subtypes, ie, low amygdala epileptogenicity values were found in lTLE in contrast with mTLE, mlTLE and TLE+.

We obtained specific results for mTLE, which was the most represented TLE subtype in our population. LA and BM tended to be more epileptogenic than other nuclei in mTLE using beta mixed model regression, however, without reaching statistical significance. LA, BM, and BL involve rather homogenous masses of cells with a cortical‐like character.^46^

LA and BL nuclei are the nuclei most susceptible to seizure‐induced damage with subsequent amygdala kindling due to the higher density of somatostatin‐containing neurons, a phenomenon that affects gamma‐aminobutyric acid‐ergic (GABAergic) transmission.^47^ In contrast with high gamma bursts in BL, cells in the LA nucleus are silent at rest but drastically increase the firing rate when activated.^48^ LA is the largest amygdala nucleus in humans, with the highest number of neurons,^35^ but the BM nucleus tends to be involved in the amygdala epileptogenicity of patients with mTLE as well. The BM nucleus receives most of these afferent connections from the BL and LA nuclei,^49^ which suggests that its epileptogenicity might be related to that of BL and LA.

### Relationship between the Amygdala Nuclei and Rhinal–Hippocampal Epileptogenicity in mTLE

We found a correlation between the epileptogenicity levels within the hippocampus and the PL nucleus in mTLE patients. These findings are in good agreement with the concept of the EZ network defined as a system of highly epileptogenic, eventually distributed but interconnected brain regions able to generate seizures which then propagate.^50^ The basolateral amygdala receives information from the entorhinal cortex and relays it to the hippocampus,^51^ partly through the PL nucleus.^52^ Indeed, PL has reciprocal connections with the hippocampus via CA1 and the subiculum.^53^ PL is characterized by a high content of immature excitatory neurons,^54^ whose differentiation to mature neurons can be enhanced in the presence of a hippocampal lesion.^55^ Due to this up‐regulation, PL could facilitate propagation of the epileptic activity from BL, LA, and BM nuclei within the amygdalo‐hippocampo‐rhinal network during mTLE seizures.

### Amygdala Nuclei Epileptogenicity: Links with Ictal Semiology

We found that epileptogenicity values were higher in BL in patients with ictal sensory phenomena and in LA in patients with autonomic phenomena, whereas lower epileptogenicity values were observed in PL in patients with cognitive phenomena. These findings remain exploratory given the complexity of anatomo‐functional networks subserving ictal semiology, which is not limited to a particular amygdala nucleus and can depend on the involvement of both the epileptogenic and the propagation networks.^56^ Yet the impact of the epileptogenicity of different amygdala nuclei may be considered as a starting point of the network dynamics within specific anatomo‐functional systems. Some speculative hypotheses on the pathophysiology of the observed semiological associations with epileptogenicity of BL and LA could be proposed. The LA nucleus receives excitatory glutamatergic inputs from thalamic and cortical sensory areas,^57^ then connects to BL to develop defensive actions.^58^ BL neurons develop increased responses that reflect potentiated sensory responses of LA neurons.^59^ Somatostatin inhibitory interneurons in BL are also innervated by cortical inputs.^59^ These mixed inhibitory and excitatory inputs to BL probably make from this nucleus the main integrative sensory hub of the basolateral complex, which could explain the observed association between its higher epileptogenicity and the presence of sensory ictal phenomena. Regarding the autonomic ictal signs in TLE, they are suggested to be mediated through activation of the hypothalamic–pituitary–adrenocortical axis, for example, via direct connections from the CEN and CMN nuclei, the main output nuclei of the amygdala, with hypothalamic nuclei,^60^ in particular the paraventricular nucleus, as well as the bed nucleus of stria terminalis. Although no association with seizure semiology could be demonstrated for CEN and CMN due to the small sample size in the present study, their activation could be enhanced through the direct or indirect excitatory input from the LA.

Regarding the cognitive phenomena, previous studies suggest that they are mediated though the rhinal–hippocampal^61^ or amygdala–rhinal interactions rather than through the direct amygdala involvement. Although the PL function is not yet well‐known, its connectivity pattern with the hippocampus, as well as its plasticity due to the presence of immature neurons as described above, underscores its role as a propagation hub within the mesial temporal network.

We did not find any association between the amygdala nuclei epileptogenicity levels and the presence of ictal affective phenomena, despite the key role of the amygdala in emotion processing and its ictal involvement previously associated with affective semiology,^62^ also reproduced by direct stimulation of the amygdala.^63^ In our cohort, only 35% of patients who experienced affective phenomena had the amygdala as the leader (EI = 1) in their seizures. Furthermore, as mentioned above, no links could be established for CEN and CMN, the main nuclei involved in emotion regulation and interaction with extratemporal limbic hubs, due to the small sample size.^64^ Other possible explanations could be that fast ictal discharge, mainly observed within the epileptogenic amygdala in the present cohort, could exhibit an inhibitory effect^65^ on the conscious perception of the emotions mediated by the amygdala.

### Limitations

This study has some limitations. First and foremost, the potential contribution of sources from the surrounding nuclei to the signal captured by the sensors in each respective nucleus cannot be formally excluded. Nonetheless, the close field organization of the amygdala^66^ and the use of bipolar referencing make such contribution less likely. The subgroups analysis helped to minimize this bias but prevented to make strong conclusions at the single nucleus level in cases of low sampling. Then, regarding the analysis of nuclei within the basolateral complex, the probability of capturing activity from the neighboring nuclei could be considered higher. This could at most reduce the significance but not fully impede the interpretation of our main results showing difference in epileptogenicity levels between these nuclei.

The implantation schemes were variable and some nuclei more rarely targeted, with high interindividual variation of sampling. The variable amount of data per nucleus at the group level could be dealt with in part using a beta mixed model, yet our statistical design remained strongly unbalanced. This is required to regroup some nuclei to analyze all the nuclei with available sampling. Nonetheless, the data were sufficient to perform precise group analyses within the basolateral complex. This spatial sampling bias could also reduce the capacity to reveal significant results for the nuclei outside the basolateral complex, as suggested above, specifically with regard to a potential link between the epileptogenicity of CEN and CMN and the affective ictal semiology. The observed correlations between the epileptogenicity levels of different mesial temporal structures, including some distinct amygdala nuclei, do not allow us to infer causality or directionality of the links between these structures. Future studies should investigate effective connectivity between the different mesial temporal subregions to better characterize their implication in TLE and correlations between amygdala nuclei involvement, the spatiotemporal electrical propagation and the emergence of ictal semiology, for example, using electrical brain stimulation.^37^ Due to the small size of the amygdala nuclei, 2 adjacent SEEG contacts could be located in different nuclei, and the corresponding SEEG signal was attributed to the location of the geometric mean of the distance between their centers. This is a methodological simplification based on previous studies from our group (eg,^67^). Monopolar referencing would not be an option to avoid this bias due to the larger volume conduction compared with bipolar referencing.^68^ The use of microelectrodes could probably allow for a more precise mapping, in particular of the smaller nuclei outside the basolateral complex.

## Conclusion

Different amygdala nuclei disclose variable epileptogenicity levels in temporal lobe epilepsy. The BL nucleus is more epileptogenic than other amygdala nuclei across all subtypes of TLE. Across the different mesial temporal structures and amygdala nuclei, the PL epileptogenicity strongly correlated with that of the hippocampus in mTLE. Future studies could investigate the amygdala connectivity using noninvasive whole‐head recordings such as magnetoencephalography.^69^, ^70^ Regarding the links with ictal semiology, the epileptogenicity values were higher in BL in patients with sensory phenomena, in LA in patients with autonomic phenomena, while the PL nucleus disclosed lower epileptogenicity in patients with cognitive phenomena.

## Author Contributions

OF, JM, FB contributed to the conception and design of the study; OF, JM, BG, FB contributed to the acquisition and analysis of data; OF, JM, SMV, MF, SL, FP, AT, MG, JPR, RC, DS, CGB, BG, FB contributed to drafting a significant portion of the manuscript or figures.

## Potential Conflict of Interest

None of the authors has conflict of interest to report.

## Supporting information


**Figure S1.** Distribution of epileptogenicity across amygdala nuclei according to the type of temporal lobe epilepsy.
**Figure S2.** Distribution of epileptogenicity according to the presence of ictal sensory phenomena.
**Figure S3.** Distribution of epileptogenicity according to the presence of ictal affective phenomena.
**Figure S4.** Distribution of epileptogenicity according to the presence of ictal cognitive phenomena.
**Figure S5.** Distribution of epileptogenicity according to the presence of ictal motor phenomena.
**Figure S6.** Distribution of epileptogenicity according to the presence of ictal autonomic phenomena.

## Data Availability

GARDEL, AnyWave software and dedicated plug‐ins (cEI) are available at https://meg.univ-amu.fr. The VEP atlas is available at https://ins-amu.fr/vep-atlas. Data that support our findings are available from the corresponding author upon reasonable request.
