# Supplementary material for: Functional Segregation of Epileptogenicity within the Human Amygdala
Source: Ann Neurol. 2026 Apr 1;99(6):1598–612. doi: 10.1002/ana.78200 (PMC13206545; doi:10.1002/ana.78200)
Supplement: Supplementary file 1 — Figure S1. Distribution of epileptogenicity across amygdala nuclei according to the type of temporal lobe epilepsy. Figure S2. Distribution of epileptogenicity according to the presence of ictal sensory phenomena. Figure S3. Distribution of epileptogenicity according to the presence of ictal affective phenomena. Figure S4. Distribution of epileptogenicity according to the presence of ictal cognitive phenomena. Figure S5. Distribution of epileptogenicity according to the presence of ictal motor phenomena. Figure S6. Distribution of epileptogenicity according to the presence of ictal autonomic phenomena. [file ANA-99-1598-s001.docx]

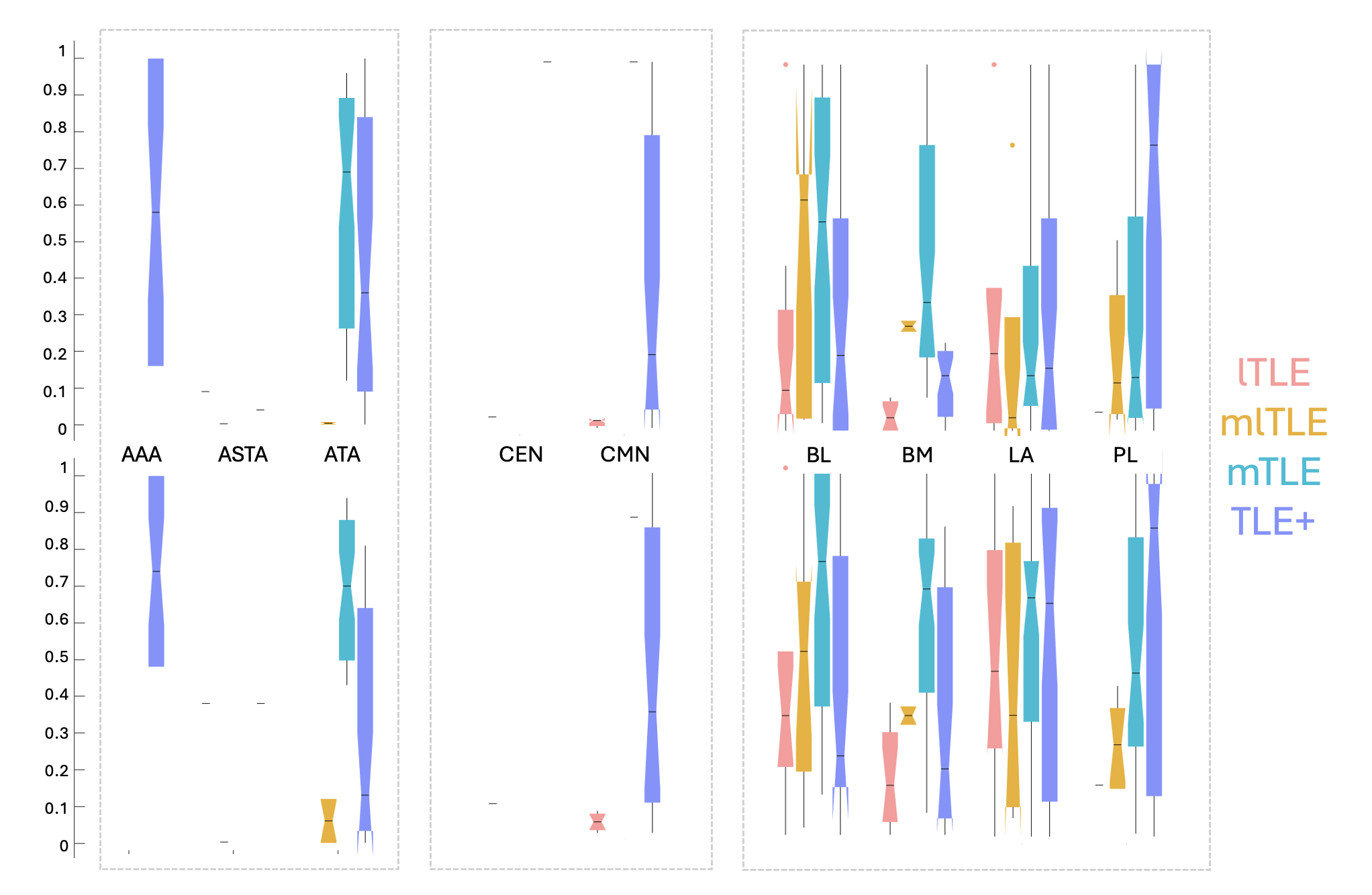


**Supplementary figure 1. Distribution of epileptogenicity across amygdala nuclei according to the type of temporal lobe epilepsy.**

Box plots of epileptogenicity index (EI, top) and connectivity epileptogenicity index (cEI, bottom) values depending on the implanted amygdala nuclei in patients with temporal lobe epilepsy (TLE): lateral TLE (pink), mesial-lateral TLE (yellow), mesial TLE (turquoise) and TLE+ (blue).

**
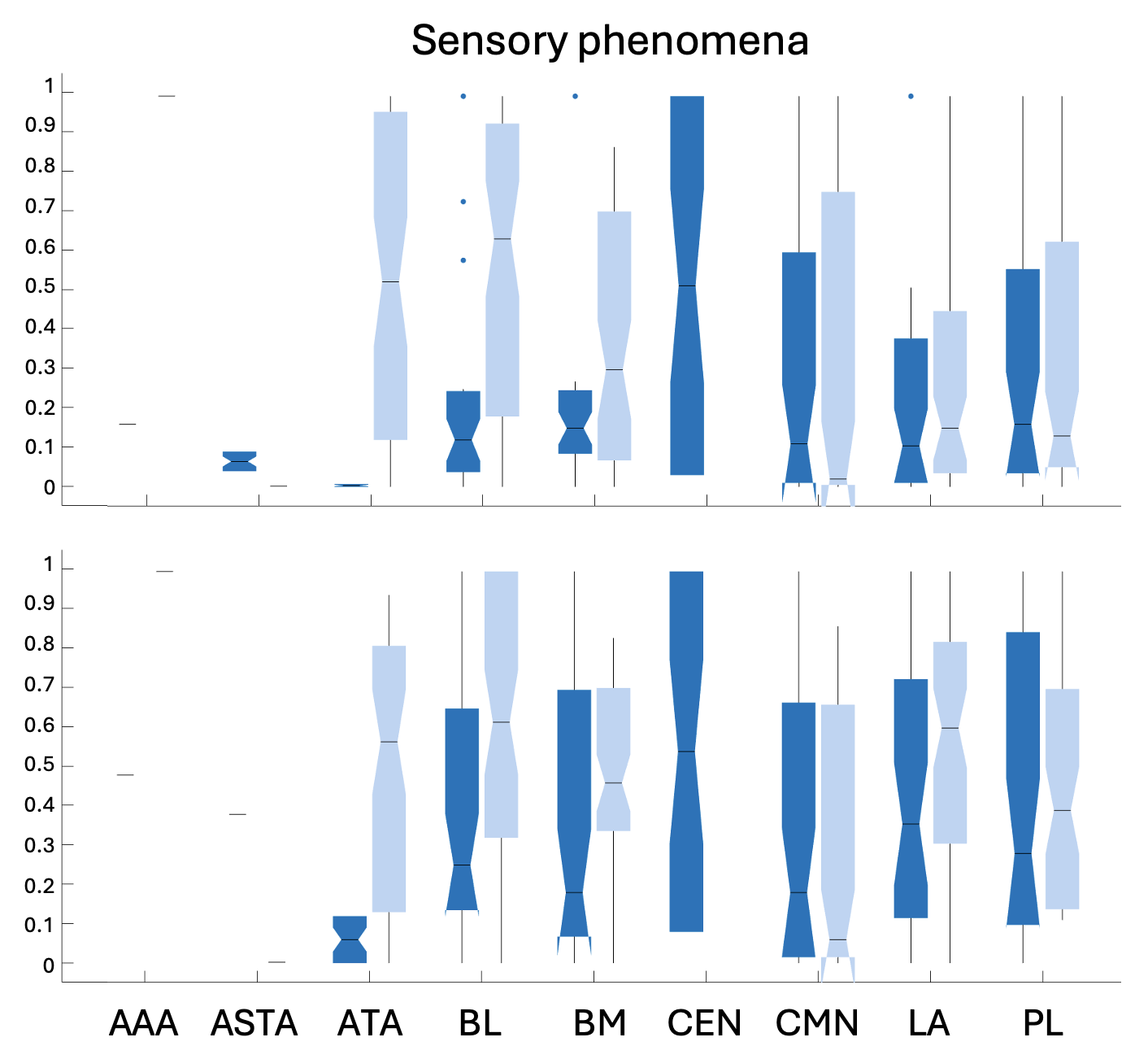
**

**Supplementary figure 2. Distribution of epileptogenicity according to the presence of ictal sensory phenomena.**

Box plots of epileptogenicity index (EI, top) and connectivity epileptogenicity index (cEI, bottom) values depending on the presence (light blue) vs. absence (dark blue) of ictal sensory phenomena. Sensory phenomena included somatosensory non-painful phenomena (n=8), somatosensory painful phenomena (n=4), auditory phenomena (n=5), vestibular phenomena (n=5), visual phenomena (n=8), gustatory phenomena (n=4), olfactory phenomena (n=2), body perception illusion (n=2). Of note, one patient can have multiple type of sensory phenomena.


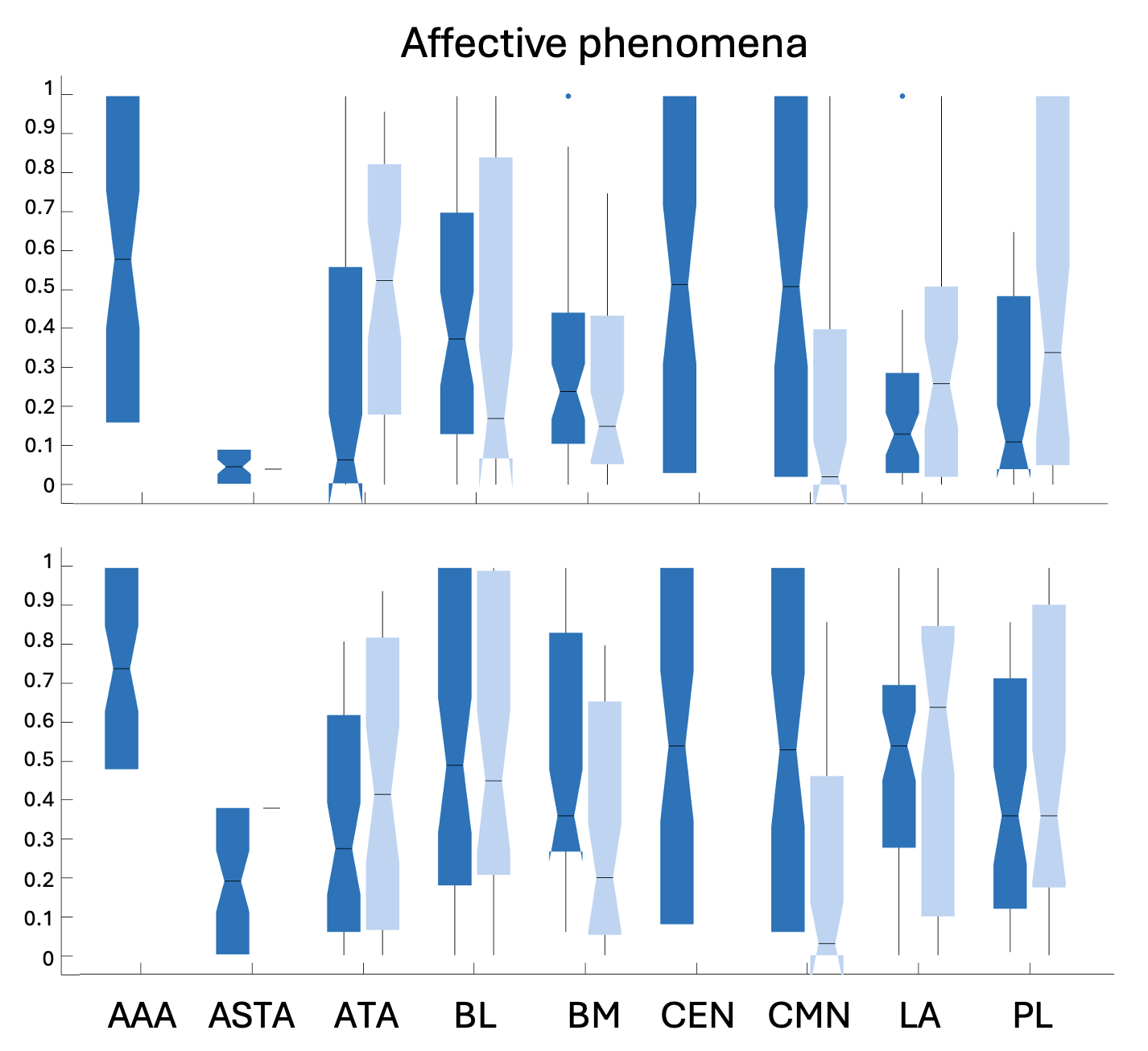


**Supplementary figure 3. Distribution of epileptogenicity according to the presence of ictal affective phenomena.**

Box plots of epileptogenicity index (EI, top) and connectivity epileptogenicity index (cEI, bottom) values depending on the presence (light blue) vs. absence (dark blue) of ictal affective phenomena. Affective phenomena were either positive (n=4; i.e., ecstatic sensation) or negative (n=19, including fear (n=5), anxiety (n=13), anger (n=1) or sadness(n=1)). Of note, one patient can have multiple type of affective phenomena.


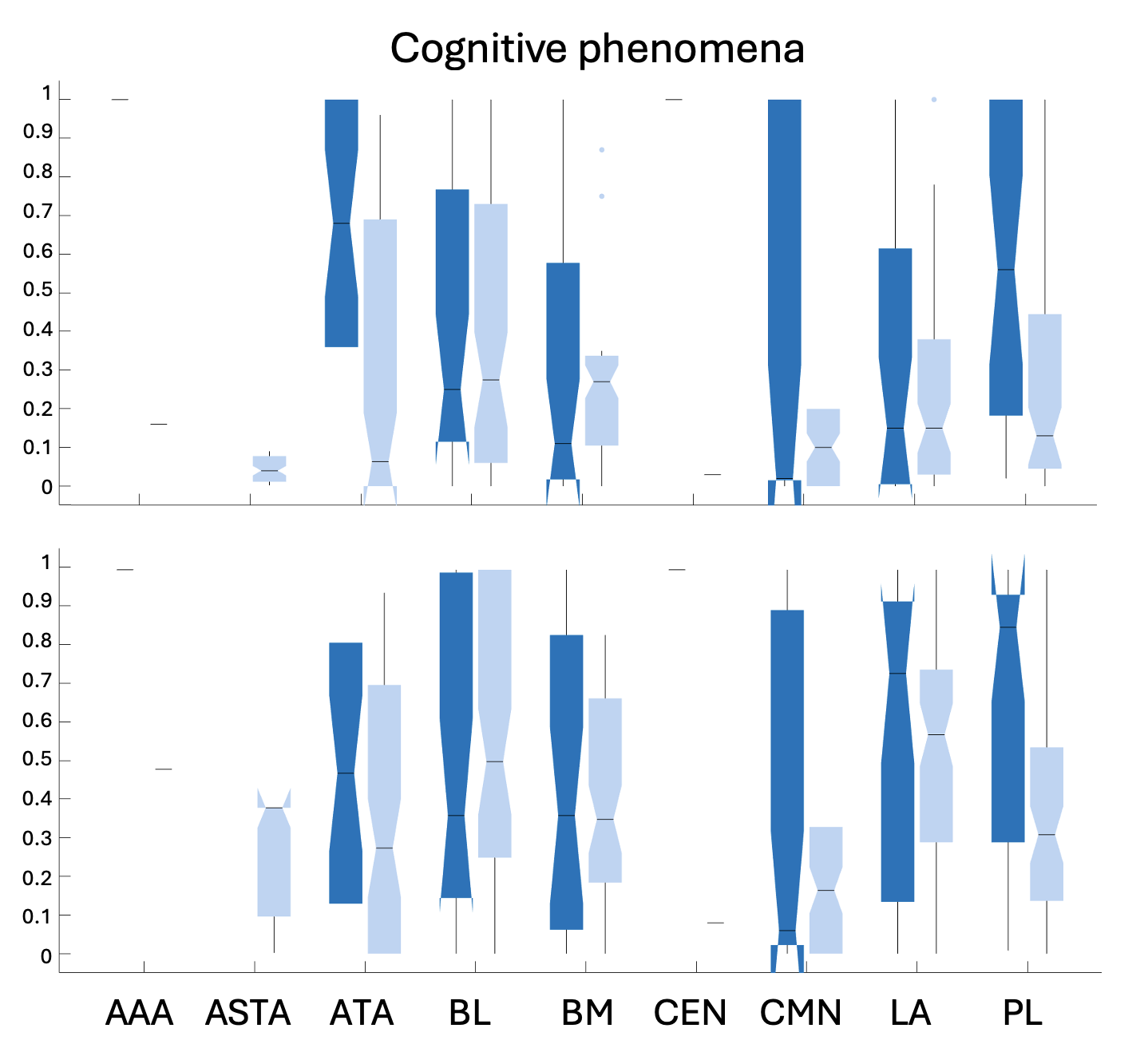


**Supplementary figure 4. Distribution of epileptogenicity according to the presence of ictal cognitive phenomena.**

Box plots of epileptogenicity index (EI, top) and connectivity epileptogenicity index (cEI, bottom) values depending on the presence (light blue) vs. absence (dark blue) of ictal cognitive phenomena. Cognitive phenomena included amnesia (n=11), aphasia (n=21), déjà vu/déjà vécu (n=12), jamais vu (n=1), disorientation (n=2), dreamy state (n=3), reminiscence (n=1). Of note, one patient can have multiple type of cognitive phenomena.


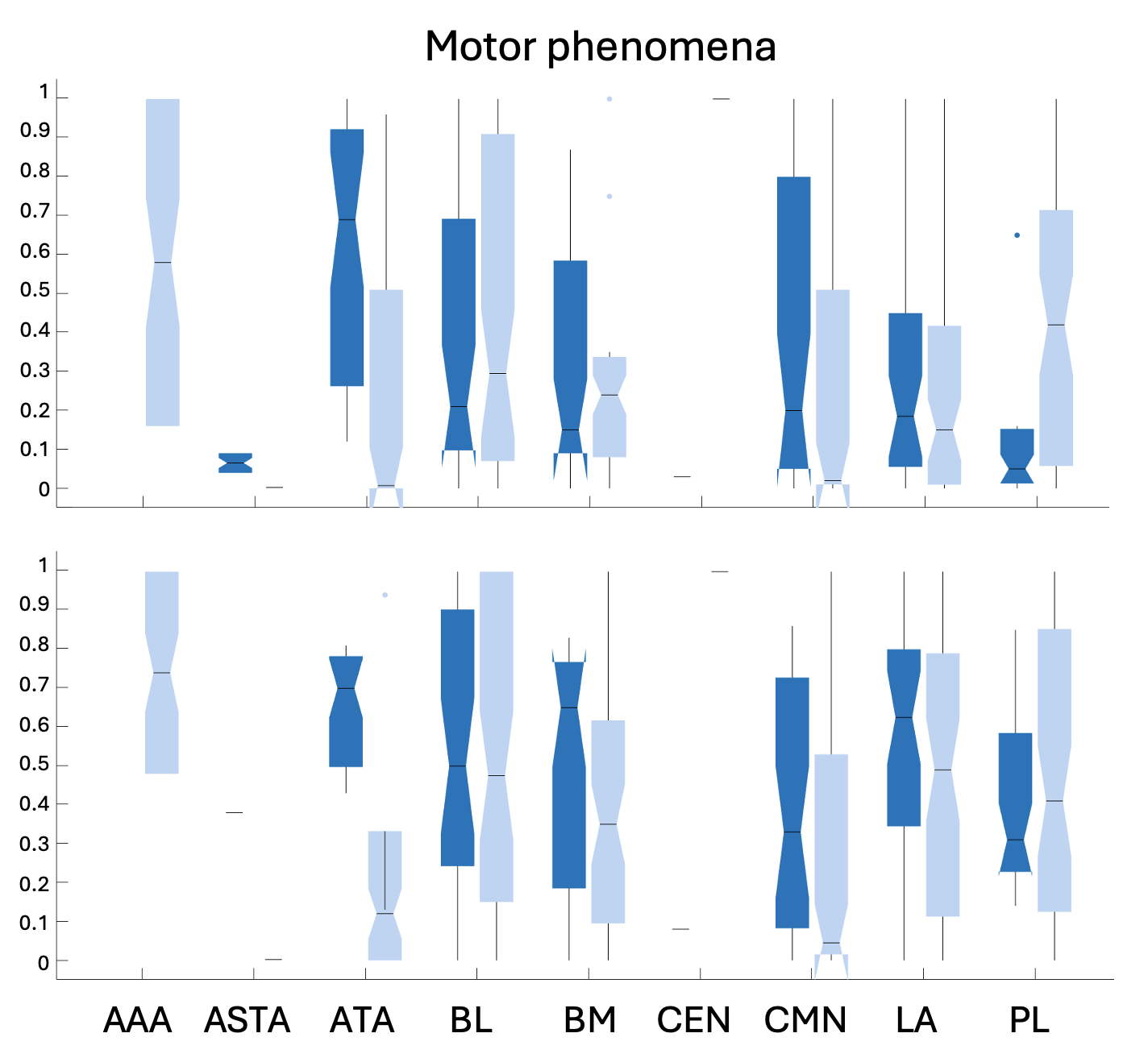


**Supplementary figure 5. Distribution of epileptogenicity according to the presence of ictal motor phenomena.**

Box plots of epileptogenicity index (EI, top) and connectivity epileptogenicity index (cEI, bottom) values depending on the presence (light blue) vs. absence (dark blue) of ictal motor phenomena. Motor phenomena mainly included oroalimentary automatisms (n=19) but could involve akinetic phenomena (n=1), focal tonic movement (n=5), gestural distal automatisms (n=9), head orientation (n=3), hyperkinetic behavior (n=2), vocal automatisms (n=1), clonic movements (n=4), eye blinking (n=1), versive (n=1), dystonic (n=2), eye deviation (n=2). Of note, one patient can have multiple type of motor phenomena.


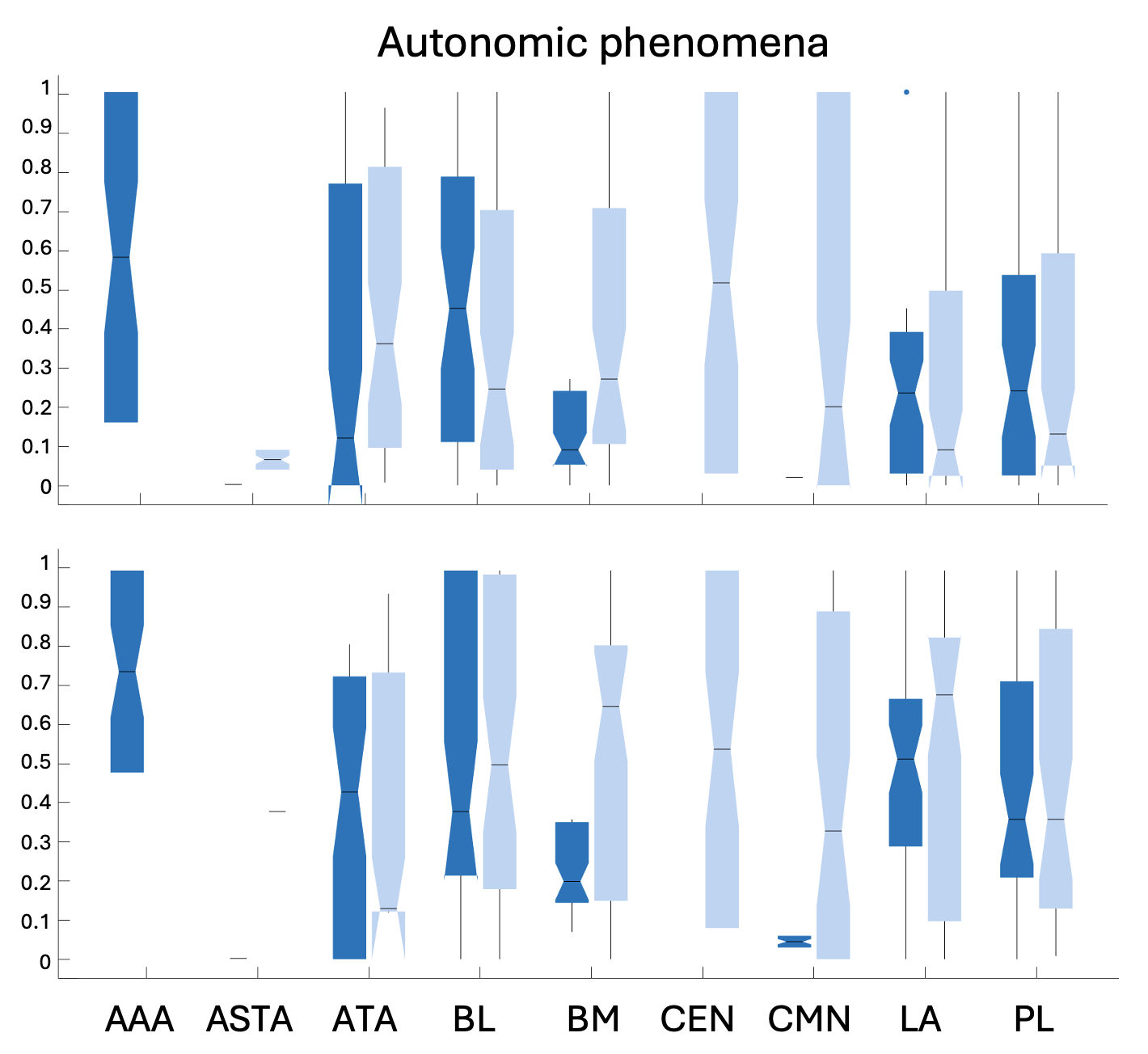


**Supplementary figure 6. Distribution of epileptogenicity according to the presence of ictal autonomic phenomena.**

Box plots of epileptogenicity index (EI, top) and connectivity epileptogenicity index (cEI, bottom) values depending on the presence (light blue) vs. absence (dark blue) of ictal autonomic phenomena. Autonomic phenomena included epigastric sensation, as the most frequent semiology of this type, and alterations of cardiac rhythm. Autonomic phenomena included epigastric sensation (n=11), flushing (n=9), hyperventilation (n=2), hypersalivation (n=4), sweating (n=2), tachycardia (n=10), hypoventilation (n=3), nausea/vomiting (n=4), borborygmi (n=1), apnea (n=1), incontinence (n=1), mydriasis (n=1). Of note, one patient can have multiple type of autonomic phenomena.
